# Factors associated with pain catastrophizing in patients with knee osteoarthritis: a cross-sectional study

**DOI:** 10.3389/fpubh.2026.1865529

**Published:** 2026-09-07

**Authors:** Lian Qiu, Yulu Yan, Guorui Li, Lin Cui, Xinyu Wang, Yingchun Wu, Xiang Yang

**Affiliations:** 1 School of Nursing, Chengdu Medical College, Chengdu, China; 2 Sichuan Provincial People’s Hospital, Chengdu, China; 3 The 944th Hospital of PLA Joint Logistics Support Force, Chengdu, China; 4 General Hospital of Western Theater Command, Chengdu, China

**Keywords:** anxiety, associated factors, knee osteoarthritis, pain catastrophizing, social support

## Abstract

**Objective:**

To understand the current status and associated factors of pain catastrophizing in patients with knee osteoarthritis.

**Method:**

Using the method of convenient sampling, 341 patients with knee osteoarthritis were recruited from 4 tertiary hospitals in Sichuan Province. From August 2023 to October 2024, data such as social demographic characteristics, Oxford Knee Score, and social support were collected through paper questionnaires.

**Result:**

The results of this study showed that the pain catastrophizing score of patients with knee osteoarthritis was (33.70 ± 10.30). The intensity of pain during activity, Oxford knee score, anxiety, and social support (*p* < 0.05) jointly explained 44.1% of the variance in pain catastrophizing.

**Conclusion:**

Patients with knee osteoarthritis have a high degree of pain catastrophizing. Clinical personnel should implement interventions in aspects such as social support for these patients to reduce the level of pain catastrophizing, optimize the pain trajectory, promote the rehabilitation process, and improve the quality of life.

## 1 Introduction

Knee osteoarthritis is a common disease among the older-adult population, with irreversible characteristics and a certain risk of disability. According to the Global Burden of Disease Study (1), as of 2020, the burden of KOA accounted for four-fifths of the total burden of osteoarthritis, with a number of affected individuals reaching 282 million. It is projected that this number will increase to 642 million by 2050. The main clinical manifestations of KOA include joint dysfunction, pain, stiffness and limited function (2). As the disease progresses, most patients also suffer from mental health problems such as depression and anxiety (3). These problems not only exacerbate the perception of pain but may also increase the risk of suicide, imposing a heavy burden on patients and society. Internationally, the direct economic cost of KOA in countries such as the United Kingdom, Canada, and Australia accounts for 1 to 2.5% of the national GDP. In the United States, KOA has become the second most expensive disease for hospitalization, accounting for 4.3% of all hospitalization costs (approximately 18.4 billion US dollars) (4). In China, the direct economic burden caused by KOA amounts to 508 million yuan, while the indirect economic burden resulting from disability-related deaths is 368.5 million yuan (5). Furthermore, the disability-adjusted life years (DALYs) rate of China’s KOA is at a relatively high level, being 1.13 times that of the United States and 1.18 times that of the global average (6). In 2022, the State Council pointed out in the “14th Five-Year National Health Plan” (7) that the aging population poses significant challenges to the disease spectrum. Strengthening the early screening and health management of key diseases in the older adults, and delaying functional decline, have become the current key development directions of medical and health work.

The concept of Pain Catastrophizing (PC) was first proposed by Spanos et al. (8) in 1979. It refers to the phenomenon where patients experience pain and develop negative emotions, making it difficult for them to shift their attention away from the pain, thereby fostering a catastrophizing cognitive tendency. Sullivan et al. (9) provided an integrated definition for it, defining PC as the negative psychological predisposition generated by an individual during actual or anticipated pain experiences, encompassing three core dimensions: rumination, amplification, and helplessness. For patients with knee osteoarthritis (KOA), PC is regarded as the most significant psychological factor influencing the degree of pain and regulating pain behavioral responses (10). Studies have shown that the incidence of pain sensitization in patients with KOA is approximately 20%, often accompanied by elevated levels of inflammatory factors and decreased pain threshold. At the same time, due to pain and limited joint mobility, patients tend to develop fear during activities and are prone to adopt an avoidance attitude, which often leads to the occurrence of PC (11, 12). Kubo et al. (13) pointed out that the incidence of pain catastrophizing among KOA patients reached 34%. Therefore, the disease occurrence mechanism and pain coping strategies of KOA patients are highly consistent with the development process of PC, suggesting that KOA patients are at high risk of concurrent PC.

Pain catastrophizing not only intensifies the patient’s perception of pain, but also has a correlation with emotional responses. Previous studies have shown (14) that the emotional state of patients with chronic pain is moderately correlated with PC, and both jointly affect the patients’ treatment compliance, medication behavior, and the risk of developing chronic pain. Studies have shown (15) that PC is closely related to hospital anxiety and depression. Patients with a higher level of pain catastrophizing are more likely to experience mental health problems such as depression and anxiety. These negative emotions will further exacerbate pain catastrophizing, and the two interact with each other, jointly delaying the recovery process of patients. Furthermore, once the level of catastrophizing pain increases, it will affect the patient’s physical functions and daily activities from both physiological and psychological perspectives, leading the patient into a negative cycle: the pain intensifies PC, which in turn affects physical and mental health, hinders the rehabilitation process, and ultimately causes the pain to further intensify (16). Therefore, effectively preventing and intervening in the pain catastrophizing of patients with KOA is of crucial significance for promoting their overall rehabilitation.

Although scholars both at home and abroad have conducted certain research on the factors related to pain catastrophizing in patients with KOA, the existing literature still lacks in the following aspects. There are significant differences among the results of various studies. For instance, some studies suggest that gender, BMI, and educational level are significantly correlated with pain catastrophizing (10, 17). However, some other studies did not find the aforementioned correlation (18); there is also no consensus regarding the moderating effects of social support and family function on PC (19, 20). This inconsistency may stem from differences in population selection, measurement tools, and control of confounding factors among various studies. Based on this, this study aims to explore the factors related to pain catastrophizing in patients with knee osteoarthritis, with the expectation of providing empirical evidence for clinical medical workers to develop individualized intervention measures and nursing management strategies.

## 2 Methods

### 2.1 Study design and participants

A cross-sectional design was adopted. This study was conducted from August 2023 to October 2024. By conducting convenient sampling, 341 patients with knee osteoarthritis were recruited from the outpatient and inpatient departments of 4 tertiary hospitals in Sichuan Province, China. Inclusion criteria: (1) The diagnosis of the disease conforms to the diagnostic criteria for knee osteoarthritis in the “Chinese Guidelines for the Diagnosis and Treatment of Osteoarthritis (2024 Edition)” (2); (2) Age ≥ 40 years (2); (3) Patients with KOA who have undergone all forms of non-surgical treatments; (4) Pain duration ≥ 3 months, with a NRS pain score ≥ 3 points (at activity); (5) Able to express themselves accurately and communicate normally. Exclusion criteria: (1) Patients with rheumatic or rheumatoid diseases, as well as other major diseases such as heart, liver, kidney, or lung disorders; (2) Patients with severe mental illness and cognitive dysfunction; (3) Patients with other chronic pains (such as cancer pain, etc.).

The sample size for this study was determined through the combined use of two methods: the estimation by the G*Power 3.1.9.7 software (21) and the rough estimation method (22). (1) Estimation using G*Power software. Based on the research design, select “*F* test” under “ANOVA: Linear multiple regression: Fixed model, *R*^2^ deviation from zero” as the statistical method. Refer to common effect size values and choose a medium effect size (Effect size *d* = 0.25). Set *α* = 0.05, test power 1 – *β* = 0.95, and include 10 predictor variables. It was calculated that at least 143 research subjects would be needed. (2) Rough Estimation Method. The estimation is conducted based on a principle of multiplying the number of independent variables by 5 to 10 (22). This study included a total of 30 independent variables. Assuming that 20% of the questionnaires are invalid, the calculated sample size range is 188 to 375 cases. Taking into account the estimation results of both methods and considering the accessibility of data collection, this study ultimately distributed 350 questionnaires, and 341 valid questionnaires were retrieved, with an effective recovery rate of 97.71%. This meets the minimum requirements of both sample size estimation methods.

### 2.2 Measurements

#### 2.2.1 General information questionnaire

The social demographic characteristics of the participants include 19 general pieces of information such as gender, age, occupation, race, place of residence, marital status, etc.

#### 2.2.2 Pain Catastrophizing Scale (PCS)

This scale was developed by Sullivan et al. (23) and is mainly used to assess the degree of catastrophizing of pain in patients, consisting of 3 dimensions: rumination (4 items), exaggeration (3 items), and helplessness (6 items). It uses a Likert 5-point scale, with a total score of 52 points. A higher score indicates a higher degree of pain catastrophizing, and a score of 30 or above is considered a high level of PC (24). The Cronbach’s α of the scale was 0.927, and the Cronbach’s α of each dimension was 0.809 (rumination), 0.839 (helplessness), and 0.768 (exaggeration). This scale has good reliability and validity for patients with KOA (10).

#### 2.2.3 Oxford Knee Score (OKS)

This scale was developed by Dawson et al. (25) and mainly assesses knee joint pain and function based on patients’ self-reports. The scale consists of 12 items, including 2 dimensions: knee joint pain (5 items) and knee joint function (7 items). Using the Likert 5-point scale, the total score ranges from 0 to 48. The higher the score, the better the patient-reported outcome. The scale has good internal consistency, with a Cronbach’s α coefficient of 0.870.

#### 2.2.4 Hospital Anxiety and Depression Scale (HADS)

This scale was developed by Zigmond and Snaith (26) and in this study, the Chinese version revised by Weifei and Junmian (27) was used. The scale consists of 14 items and includes two subscales: anxiety (7 items) and depression (7 items). Each item is scored on a 4-point scale ranging from 0 to 3. When the scores of the depression or anxiety subscales are both ≥ 8 points, it can be determined that the patient has symptoms of anxiety or depression. The Cronbach α coefficients of the Chinese versions of the anxiety and depression scales are (26) 0.762 and 0.787, respectively.

#### 2.2.5 Social Support Rating Scale (SSRS)

This scale was developed by Xiao Shuiyu in 1986 (28), consisting of three dimensions and a total of 10 items: objective support (3 items), social support utilization (3 items), and subjective support (4 items). The total score ranges from 12 to 66, among which the subjective support score ranges from 8 to 32, the objective support score ranges from 1 to 22, and the support utilization score ranges from 3 to 12. The higher the score, the higher the level of social support. The Cronbach α coefficient is 0.806.

#### 2.2.6 Family adaptation, partnership, growth, affection, resolve (APGAR)

This tool was developed by Smilkstein et al. (29) to measure the satisfaction of family members with the family’s functions. Each item is rated as “rarely,” “sometimes” or “often,” and the score for each item ranges from 0 to 2. The APGAR total score ranges from 0 to 10. A score of 7 to 10 indicates good function, a score of 4 to 6 indicates severely impaired function, and a score of 0 to 3 indicates severely impaired function. The Cronbach’s alpha coefficient is 0.83, demonstrating good reliability and validity.

### 2.3 Data collection and consent for publication

Before data collection, all investigators underwent standardized training to ensure the uniformity of the interview procedures. Participants provided written informed consent. Considering that there might be different levels of cultural background and ages among the research subjects, before clinical treatment, the researchers collected data through face-to-face interviews conducted on the day of admission. The questionnaire is managed and filled out by the investigators on behalf of the participants. After completing the questionnaire, the participants will immediately review each one item by item to clarify ambiguous responses and make on-site corrections for errors before the final collection. All data are entered independently twice using two separate computers within 48 h.

### 2.4 Data analyses

All data analyses were conducted using version 9.4 for Windows (SAS Institute, Inc., Cary, NC, United States) and Version 3.4.3 for data management and analysis. The count data were described using frequency and percentage. Measurements that followed a normal distribution were presented as mean ± standard deviation, while measurements with a skewed distribution were described using the median and interquartile range (P_25_, P_75_). We used independent sample *t*-tests or analysis of variance to determine whether there were differences in pain catastrophizing among patients with KOA from different sociodemographic backgrounds. Results with *p* < 0.05 were considered statistically significant; Pearson correlation analysis and Spearman rank correlation coefficient analysis were employed to examine the correlations. Finally, using hierarchical regression analysis to identify the relevant factors contributing to the pain catastrophizing in patients with KOA (Knee Osteoarthritis). No missing data were present in the final dataset, as all questionnaires were reviewed and completed on-site by investigators.

## 3 Outcome

A total of 350 questionnaires were distributed in this study, and 341 were finally retrieved, with an effective recovery rate of 97.71%. Table 1 presents the demographic variables of the participants. 68.1% were female, and those aged 40–65 accounted for 48.9%. The Han Chinese accounted for 96.1%; there were more rural residents; among the marital statuses, 81.2% were married; the main way of seeking medical treatment was outpatient visits. There were 299 patients who did not use opioid drugs; 291 patients had not received joint cavity injection drugs; more than half of the participants had a fixed caregiver, and a common BMI value for the patients was ≤24 kg/m^2^. There were 163 cases with 1 to 2 comorbidities. The average NRS score of patients with knee osteoarthritis during activity was (5.49 ± 1.75) points, mainly manifested as moderate pain. Due to the fact that the farming population accounted for a large proportion (58.4%) in this study, the monthly income of most patient families was not fixed (66.5%), and the main medical payment method was the new rural cooperative medical care system. The educational level was mainly at the primary school level or below; the majority did not smoke or drink; the duration of pain was mostly less than 1 year (Table 1).

**Table 1:** Comparison of pain catastrophizing by different sociodemographic characteristics (*n* = 341).

Sociodemographic characteristics	Group	*n*(%)	*M* ± *SD*	*t*/*F*	*P*	Post-event comparison
Gender	Man	109(31.9)	33.67 ± 10.47	−0.034	0.973	
	Woman	232(68.1)	33.71 ± 10.24			
Age group	40 ~ 65^a^	167(48.9)	31.65 ± 10.66	6.907	0.001	b > a
	66 ~ 79^b^	150(43.9)	35.85 ± 9.72			
	≥80^c^	24(7.0)	34.50 ± 11.33			
Education	Primary school or below	192(56.3)	34.88 ± 9.346	2.032	0.109	
	Junior high school	90(26.4)	31.98 ± 11.82			
	High school or vocational school	34(10.0)	32.94 ± 10.29			
	College or above	25(7.3)	31.84 ± 10.79			
Residence	Town/City	149(43.6)	32.68 ± 10.29	−1.604	0.110	
	Rural	192(56.4)	34.48 ± 10.26			
Marital status	Unmarried	64(18.8)	34.11 ± 10.98	−0.337	0.737	
	Married	277(81.2)	33.60 ± 10.15			
Average monthly household income per capita (yuan)	Not fixed^a^	227(66.5)	34.30 ± 10.29	3.373	0.035	a > c, b > c
	<3000^b^	66(19.4)	34.21 ± 9.22			
	≥3000^c^	48(14.1)	30.15 ± 11.20			
Occupation	Retired personnel	80(23.5)	32.64 ± 10.00	1.224	0.301	
	In-service	52(15.2)	32.69 ± 9.68			
	Agriculture	199(58.4)	34.55 ± 10.48			
	Unemployed	10(2.9)	30.50 ± 11.68			
Ethnicity	Han Chinese	328(96.1)	33.50 ± 10.36	−1.789	0.074	
	Others	13(3.9)	38.69 ± 6.93			
Method of admission	Outpatient Clinic	314(92.1)	33.40 ± 10.25	1.830	0.077	
	Emergency	27(7.9)	37.19 ± 10.32			
Smoking/Drinking	None	270(79.1)	33.45 ± 10.48	0.648	0.585	
	Smoking	16(4.1)	37.13 ± 11.05			
	Drinking	24(7.1)	33.88 ± 9.76			
	Both	31(9.1)	33.94 ± 8.68			
Comorbidities	0	142(41.6)	32.99 ± 10.27	1.293	0.276	
	1 ~ 2	163(47.8)	33.80 ± 10.42			
	≥3	36(10.6)	36.06 ± 9.71			
Using opioid drugs	Yes	42(12.3)	37.12 ± 9.68	2.314	0.021	
	No	299(87.7)	33.22 ± 10.30			
Intra-articular injection	Yes	50(14.7)	35.30 ± 10.22	1.199	0.235	
	No	291(85.3)	33.42 ± 10.30			
Caregiver	Yes	222(65.1)	34.66 ± 10.51	2.378	0.018	
	No	119(34.9)	31.90 ± 9.66			
BMI (kg/m^2^)	<24	157(46.0)	33.36 ± 10.05	0.950	0.388	
	24.0 ~ 27.9	129(37.8)	34.61 ± 9.85			
	≥28	55(16.1)	32.53 ± 11.90			
NRS score (activity)	=3^a^	60(17.6)	27.22 ± 13.76	47.123	<0.001	c > b > a
	4 ~ 6^b^	178(52.2)	31.94 ± 8.55			
	7 ~ 10^c^	103(30.2)	40.51 ± 6.32			
Duration of pain (years)	<1^a^	121(35.5)	31.80 ± 11.54	3.421	0.018	d > a
	1 ~ 5^b^	104(30.5)	33.77 ± 9.11			
	6 ~ 10^c^	38(11.1)	33.71 ± 8.26			
	>10^d^	78(22.9)	36.54 ± 10.13			

*The variance analysis was conducted using the Welch test, and the *post hoc* comparisons were performed using Tamhane’s T2 test; a, b, c represent different categories, set group a as the reference.

### 3.1 Baseline information of PCS, OKS, HADS, SSRS and APGAR for patients with KOA

Overall, 241 patients scored above 30 on the Pain Catastrophizing Scale (PCS), indicating that 70.6% of the KOA patients exhibited a high level of pain catastrophizing. In this study, 133 participants (39.0%) were identified as anxious, and 67 participants (19.6%) were found to be depressed. Most patients reported a moderate level of social support and received good family care (Table 2).

**Table 2:** Scores of PCS, OKS, HADS, SSRS and APGAR for KOA patients (*n* = 341).

Characteristics	*M* ± *SD*	Range	Actual range	
			Min	Max
Pain Catastrophizing Scale	33.70 ± 10.30	0 ~ 52	0	52
Rumination	10.62 ± 3.66	0 ~ 16	0	16
Helplessness	15.28 ± 4.84	0 ~ 24	0	24
Magnification	7.59 ± 2.44	0 ~ 12	0	12
Oxford Knee Score	18.47 ± 7.97	0 ~ 48	0	48
Knee pain	6.53 ± 3.48	0 ~ 20	0	20
Function	11.94 ± 5.13	0 ~ 28	0	28
Hospital Anxiety and Depression Scale	11.56 ± 5.74	0 ~ 42	0	31
Anxiety	6.73 ± 3.08	0 ~ 21	0	20
Depression	4.82 ± 3.35	0 ~ 21	0	18
Social Support Rating Scale	30.10 ± 6.17	12 ~ 66	17	45
Subjective support	16.35 ± 3.60	8 ~ 32	8	24
Objective support	7.20 ± 1.61	1 ~ 22	2	12
Utilization of social support	6.58 ± 2.62	3 ~ 12	3	12
Family APGAR Index Scale	6.87 ± 2.17	0 ~ 10	0	10

### 3.2 Correlation analysis of pain catastrophizing in patients with knee osteoarthritis

The pain catastrophizing score was positively correlated with anxiety and depression levels (*p* < 0.01); it was negatively correlated with OKS score, the level of social support received by the patients, and the degree of family care (*p* < 0.01). Most of the correlation values were between 0.2 and 0.5, indicating a moderate correlation between pain catastrophizing and OKS, anxiety, depression levels, social support level, and family care degree (Table 3).

**Table 3:** Correlation between pain catastrophizing and independent variables (*n* = 341).

Items	Pain catastrophizing	Oxford knee score	Anxiety	Depression	Social support	Family care
Pain catastrophizing	1	–	–	–	–	–
Oxford Knee Score	−0.439**	1	–	–	–	–
Anxiety	0.539**	−0.419**	1	–	–	–
Depression	0.422**	−0.422**	0.595**	1	–	–
Social Support	−0.343**	0.242**	−0.297**	−0.496**	1	–
Family Care	−0.218**	0.096	−0.217**	−0.361**	0.510**	1

**p < 0.01.

### 3.3 Factors related to pain catastrophizing

Hierarchical regression analysis was employed to explore the factors related to pain catastrophizing in patients with knee osteoarthritis. The D-W statistic is 1.870, indicating that the data are independent. The residual scatter plot shows points distributed symmetrically around zero, suggesting that the homoscedasticity assumption is met. The residual P–P plot and residual histogram conform to a normal distribution. The VIF value is lower than 5 and the tolerance value is above 0.2, indicating that there is no serious multicollinearity problem among the variables in the regression model. In Model 1, we used the pain catastrophizing items as the dependent variable and selected variables such as age, average monthly family income, whether using opioid drugs, whether having a caregiver, duration of pain, and NRS Scores (activity) as the independent variables for hierarchical regression analysis, which were statistically significant. After controlling for the general characteristics, we included variables such as Oxford Knee Score, Anxiety, Social Support, Family Care and Depression as independent variables in Model 2. The results of the regression model indicated that NRS Scores (activity), Anxiety, Oxford Knee Score, and Social Support were the relevant factors for pain catastrophizing (Table 4).

**Table 4:** Factors related to the catastrophizing of pain in patients with knee osteoarthritis (*n* = 341).

Variables	Model 1								Model 2							
	*B*	SE	*β*	*t(P)*	Collinear statistics		95%CI		*B*	SE	*β*	*t(P)*	Collinear statistics		95%CI	
					Tolerance	VIF	Lower limit	Upper limit				Tolerance	Tolerance	VIF	Lower limit	Upper limit
(Constent)	2.375	0.284		8.377(<0.001)			1.817	2.933	2.884	0.370		7.799(<0.001)			2.157	3.611
Age group																
66 ~ 79	0.223	0.080	0.140	2.785(0.006)	0.868	1.152	0.065	0.380	0.049	0.073	0.031	0.674(0.501)	0.779	1.284	−0.095	0.193
≥80	0.274	0.153	0.089	1.797(0.073)	0.900	1.112	−0.026	0.574	0.061	0.138	0.020	0.444(0.658)	0.830	1.205	−0.210	0.332
D. Average monthly household income per capita (yuan)																
<3,000	0.043	0.097	0.022	0.444(0.657)	0.932	1.073	−0.148	0.234	0.047	0.085	0.023	0.551(0.582)	0.921	1.086	−0.120	0.213
≥3,000	−0.220	0.116	−0.097	−1.905(0.058)	0.849	1.178	−0.447	0.007	−0.125	0.101	−0.055	−1.241(0.215)	0.837	1.195	−0.324	0.073
Using opioid drugs	−0.229	0.116	−0.096	−1.979(0.049)	0.947	1.056	−0.457	−0.001	−0.080	0.101	−0.033	−0.793(0.428)	0.926	1.080	−0.280	0.119
Caregiver	0.013	0.084	−0.008	−0.154(0.877)	0.848	1.179	−0.153	0.179	−0.013	0.075	−0.008	−0.178(0.859)	0.806	1.241	−0.161	0.134
D. NRS score (Activity)																
4 ~ 6	0.346	0.103	0.219	3.348(0.001)	0.513	1.949	0.143	0.550	0.272	0.092	0.172	2.972(0.003)	0.491	2.038	0.092	0.453
7 ~ 10	0.991	0.114	0.576	8.720(<0.001)	0.503	1.988	0.768	1.215	0.667	0.105	0.387	6.367(<0.001)	0.445	2.249	0.461	0.873
D. Duration of pain (Years)																
1 ~ 5	0.065	0.093	0.038	0.696(0.487)	0.748	1.336	−0.118	0.248	0.113	0.081	0.066	1.401(0.162)	0.743	1.346	−0.046	0.272
6 ~ 10	−0.046	0.130	−0.018	−0.357(0.721)	0.820	1.219	−0.302	0.209	−0.046	0.113	−0.018	−0.405(0.686)	0.809	1.236	−0.269	0.177
>10	0.175	0.105	0.093	1.667(0.097)	0.704	1.421	−0.032	0.382	0.155	0.091	0.082	1.695(0.091)	0.698	1.433	−0.025	0.335
Oxford Knee Score									−0.029	0.007	−0.190	−4.008(<0.001)	0.734	1.362	−0.044	−0.015
Depression									−0.050	0.100	−0.030	−0.496(0.620)	0.451	2.218	−0.247	0.147
Anxiety									0.577	0.096	0.320	6.021(<0.001)	0.582	1.719	0.389	0.766
Social Support									−0.189	0.068	−0.147	−2.792(0.006)	0.591	1.693	−0.322	−0.056
Family Care									−0.022	0.091	−0.012	−0.245(0.807)	0.663	1.509	−0.201	0.157
	*R*^2^ = 0.279, adjusted *R*^2^ = 0.255, △*R*^2^ = 0.279 F = 11.570 (*p* < 0.001), D-W = 1.870								*R*^2^ = 0.467, adjusted *R*^2^ = 0.441, △*R*^2^ = 0.188 *F* = 17.735 (*p* < 0.001), D-W = 1.870							

D. As the reference group for the dummy variable. Age group (40 ~ 65), Average monthly household income per capita (Not fixed), NRS score (Activity) (=3), Duration of pain (Years) (<1).

## 4 Discussion

The level of pain catastrophizing in patients with knee osteoarthritis has a direct impact on their physical and mental health. The intensity of pain during activities, knee joint function, anxiety, and social support are all related to the level of pain catastrophizing in patients with knee osteoarthritis. The final model explained 44.1% of the total variance in pain catastrophizing. The addition of the second layer of psychosocial factors brought about a significant 18.8% increase in explanatory power. These findings can provide empirical evidence for the formulation of personalized intervention plans and the optimization of clinical nursing strategies, thereby effectively reducing the level of pain catastrophizing in patients, improving the trajectory of pain development, promoting the rehabilitation process, and enhancing overall quality of life.

### 4.1 Pain Catastrophizing Scale (PCS)

In our study, the pain catastrophizing score of patients with knee osteoarthritis was 33.70 ± 10.30, indicating that the severity of pain catastrophizing in these patients was high. This result is largely consistent with previous studies, but the severity is higher than that in previous studies that focused on patients undergoing surgery or conservative treatment (13, 30, 31). This might be due to the fact that the patients included in our study were mainly those who visited the hospital for the first time. These patients were mostly experiencing their first onset of the disease, had a heavy disease burden, and had poor pain tolerance (32); secondly, a relatively high proportion of the subjects were from rural areas. Their disease perception ability was generally low, and they tended to seek medical treatment only when their symptoms were severe, which led to a faster disease progression and higher pain levels (33). Furthermore, the subjects of this study were all patients with KOA who had undergone all forms of non-surgical treatment. Therefore, some of these patients had received traditional Chinese medicine treatment before admission, such as basic painkillers or acupuncture, but the pain relief effect was limited (34). Therefore, medical staff should pay attention to the problem of pain catastrophizing in such patients. Especially in the first visit, in rural areas, and in patients with knee osteoarthritis who have not yet received systemic treatment, they should identify their psychological and pain conditions early and conduct targeted assessment and intervention to alleviate the pain experience and improve the prognosis of the disease.

### 4.2 The factors associated with pain catastrophizing in patients with knee osteoarthritis

#### 4.2.1 The intensity of pain during the activity and its correlation with pain catastrophizing

Our research results show that the intensity and duration of pain during activities are positively correlated with pain catastrophizing (*p* < 0.01), which is consistent with the conclusion of Kubo et al. (13). Pain catastrophizing is essentially a negative psychological reaction of an individual to pain. For patients with KOA, their pain is often a complex combination of inflammatory, nociceptive, and neuropathic pain (35). Therefore, the higher the pain intensity, the stronger the tendency of patients to respond negatively to pain, and the frequency of pain catastrophizing also increases accordingly. Based on the fear-avoidance model’s theoretical framework (36), the aforementioned association can be understood from both cognitive and behavioral perspectives. At the cognitive level, when patients are in an active state, the reduction of the pain perception threshold leads to an enhanced subjective pain experience. Patients tend to interpret the pain signals as threatening, develop rumination thinking, and subsequently experience amplified pain sensations. At the behavioral level, it manifests as a negative coping pattern of activity avoidance, which may trigger secondary functional disorders such as atrophy of unused muscles. And studies have shown (37) that if systematic pain management intervention is lacking within 6 weeks after the onset of pain, the risk of patients developing pain catastrophizing will increase to 1.8 times the baseline level. Therefore, timely and effective pain control is a crucial entry point to break the vicious cycle of pain catastrophizing. Clinically, the focus should be on reducing pain. Through multimodal analgesic strategies, pain symptoms should be controlled promptly, and the pain intensity should be reduced to an acceptable range, which can effectively prevent the occurrence and development of pain catastrophizing.

#### 4.2.2 The correlation between knee joint function and pain catastrophizing

The results of this study show that there is a negative correlation between the knee joint function score and the pain catastrophizing score of patients with knee osteoarthritis (*p* < 0.01), suggesting that the function of the knee joint is closely associated with the risk of developing pain catastrophizing. These results are consistent with previous findings (38). The functional condition of the knee joint increases the risk rate of PC through avoidance behaviors and alerting pain. Patients with knee joint dysfunction will exhibit abnormal compensatory gait, limitations in daily activities, and frequent knee pain symptoms (36). Moreover, these patients often lack the ability to effectively manage pain symptoms, which exacerbates helpless cognition and avoidance of daily life, entering a vicious cycle of “exercise – pain – avoidance”. During this cycle, the probability of PC significantly increases. PC can cause patients to be highly alert and fearful of pain, thus avoiding activities that may trigger pain. Although this avoidance behavior can alleviate pain temporarily, in the long run, it will lead to muscle atrophy, joint stiffness and psychological problems, further exacerbating PC (39). Therefore, clinical staff should focus on functional exercises for individuals with a high level of pain catastrophizing. Compared to simple exercise therapy, exercise combined with cognitive therapy can reduce the level of pain catastrophizing and improve the effectiveness of exercise intervention (40). Studies have shown (41) that pain neuroscience education combined with physical therapy, through graded exposure exercises, can reduce the sensitivity of the nervous system, break the pain memory triggered by exercise, and has a good therapeutic effect on KOA patients with high levels of pain catastrophizing. In conclusion, before implementing exercise therapy and rehabilitation training for KOA patients, their level of pain catastrophizing should be evaluated. For those with a high level of this, a cognitive-motor combined intervention strategy is recommended. In the future, clinical research should further explore cognitive therapies and intervention measures for KOA patients with high levels of pain catastrophizing, and promote the implementation of personalized treatment processes.

### 4.3 The correlation between psychological factors and pain catastrophizing

In the correlation analysis, although depression was significantly positively correlated with pain catastrophizing, in the final model of hierarchical regression, its effect was no longer significant. The collinearity diagnosis also indicated that this result was not caused by multiple collinearity. Therefore, we believe that it might be due to other variables with stronger explanatory power in the model masking its independent effect, or there might be an intermediary effect, etc. Rogers et al. (42) conducted a study showing that pain catastrophizing was positively correlated with anxiety and depression. Our research results are largely consistent with theirs and are also in line with previous studies on chronic pain populations (43). Pain catastrophizing can directly affect an individual’s behavioral performance, functional status, and quality of life by regulating the functions of the central nervous system (44). Anxiety is mainly characterized by excessive worry about the future, while depression is defined by sadness and despair as its core features. Both involve negative expectations for the future and are closely related and mutually influential (45). Due to the influence of diseases, patients with depression often have a negative perception of pain. This pessimistic attitude may enhance their sensitivity to pain and even unconsciously amplify the pain experience, thereby further increasing the level of pain catastrophizing (46). At the same time, patients who are in a chronic pain state for a long time are prone to induce anxiety, resulting in a vicious cycle of interaction and mutual aggravation between pain catastrophizing and anxiety, as well as depression. Therefore, for patients with anxiety or depression symptoms, a low-intensity functional exercise plan can be designed to alleviate anxiety (47). This can reduce the catastrophizing of pain, enhance the patients’ autonomy in functional exercise, and promote their recovery process.

### 4.4 The correlation between social support and pain catastrophizing

In our study, there was a negative correlation between social support and family function of patients with knee osteoarthritis and pain catastrophizing (*p* < 0.01), which was consistent with the research results of Matthias (20) and others. The PC-based joint response model (48) suggests that the catastrophizing behavior of patients with chronic pain might be a form of seeking social support, that is, by exaggerating negative emotions to obtain sympathy and support within intimate relationships. Specifically, high levels of social support mean that patients have more external resources at their disposal, allowing them to express their pain more directly. This is beneficial in reducing the sense of helplessness and rumination in the pain condition (PC). Meanwhile, when adequate social support is provided, it can alleviate the psychological distress of patients, reduce their excessive threat perception of pain, and thereby decrease the risk of the occurrence of PC. In the management of chronic diseases such as KOA, high levels of social support not only encourage patients to adhere to healthy self-management, but also help reduce invasive thoughts related to the disease. Based on this, in clinical practice, the construction of a social support system should be regarded as a key aspect of chronic pain management. Through a systematic assessment of the patient’s social support network, a targeted support system should be constructed and optimized to enhance the patient’s self-management ability, and to provide emotional and behavioral support, thereby effectively preventing and alleviating the catastrophizing of pain.

### 4.5 Limitations

This study has the following limitations: firstly, this study only included patients from 4 tertiary hospitals in Sichuan Province. The sample representation was rather limited. Therefore, future research should adopt a multi-center stratified sampling strategy and include medical institutions from different regions and at different levels as much as possible to enhance the general applicability of the research conclusions. Secondly, this study adopted a cross-sectional design and lacked long-term follow-up data. Therefore, it can only reveal the relevant factors and relationships of pain catastrophizing in knee osteoarthritis, but cannot clearly determine the causal direction and action pathway. In the future, large-sample cohort studies or experimental designs that control confounding variables can be conducted to further verify the impact mechanism of these factors on delayed medical treatment, in order to make up for the shortcomings of the current study. Thirdly, this study did not conduct descriptive stratification of the treatment types, which may have prevented a thorough control and comparison of the effects of different treatment plans on the level of pain catastrophizing. As a result, it may have affected the accuracy and interpretability of the results to some extent. Future studies should include treatment types as a stratification variable to further explore their potential roles in the formation and evolution of pain catastrophizing.

## 5 Conclusion

This study reflects the current situation of pain catastrophizing among patients with knee osteoarthritis and explores the related factors of pain catastrophizing. In this study, the intensity of pain during activities, knee joint function, anxiety, and social support were the relevant factors for pain catastrophizing in patients with knee osteoarthritis. In this study, the degree of pain catastrophizing among patients with knee osteoarthritis was generally high, which requires clinical attention. Medical workers should actively adopt comprehensive intervention measures, including providing psychological counseling, effectively alleviating patients’ pain, and systematically establishing a social support system, in order to reduce the level of pain catastrophizing among patients and improve their physical and mental conditions.

## Data Availability

The original data on which the conclusions of this article are based can be provided by the author free of charge upon reasonable request.

## References

[1] GBD 2021 Osteoarthritis Collaborators. Global, regional, and national burden of osteoarthritis, 1990-2020 and projections to 2050: a systematic analysis for the global burden of disease study 2021. Lancet Rheumatol. (2023) 5:e508–22. doi: 10.1016/S2665-9913(23)00163-7 PMC10477960 37675071

[2] Association OCOC. Guideline for diagnosis and non-surgical treatment of early-stage knee osteoarthritis (2024 edition). Zhonghua Yi Xue Za Zhi. (2024) 104:2895–909. doi: 10.3760/CMA.J.CN112137-20240503-01035 39118337

[3] Hai W Jian P Bo C YuLong C Hongsheng Z. Investigation on anxiety and depression in patients with knee osteoarthritis. Chin J Pharm Inf. (2018) 15:47–50.

[4] Leifer VP Katz JN Losina E. The burden of oa-health services and economics. Osteoarthr Cartil. (2022) 30:10–6. doi: 10.1016/j.joca.2021.05.007, 34023527 PMC8605034

[5] Yang G Wang J Liu Y Lu H He L Ma C . Burden of knee osteoarthritis in 204 countries and territories, 1990-2019: results from the global burden of disease study 2019. Arthritis Care Res. (2023) 75:2489–500. doi: 10.1002/acr.25158, 37221154

[6] Xiaocheng F Daozhang C Xinglei Y Shijie Z Wen J. Analysis of the current situation and trends of knee osteoarthritis disease burden in China based on GBD big data. Mod Prev Med. (2022) 49:1753–60.

[7] China to promote national health during 14th five-year plan (2022). Available online at: https://english.www.gov.cn/policies/latestreleases/202205/20/content_WS62874295c6d02e533532b0bd.html (Accessed April, 2026).

[8] Spanos NP Radtke-Bodorik HL Ferguson JD Jones B. The effects of hypnotic susceptibility, suggestions for analgesia, and the utilization of cognitive strategies on the reduction of pain. J Abnorm Psychol. (1979) 88:282–92. doi: 10.1037//0021-843x.88.3.282, 500957

[9] Sullivan MJ Thorn B Haythornthwaite JA Keefe F Martin M Bradley LA . Theoretical perspectives on the relation between catastrophizing and pain. Clin J Pain. (2001) 17:52–64. doi: 10.1097/00002508-200103000-00008, 11289089

[10] Ong WJ Kwan YH Lim ZY Thumboo J Yeo SJ Yeo W . Measurement properties of pain catastrophizing scale in patients with knee osteoarthritis. Clin Rheumatol. (2021) 40:295–301. doi: 10.1007/s10067-020-05163-8, 32519053

[11] Previtali D Capone G Marchettini P Candrian C Zaffagnini S Filardo G. High prevalence of pain sensitization in knee osteoarthritis: a meta-analysis with meta-regression. Cartilage. (2022) 13:788765230. doi: 10.1177/19476035221087698, 35356833 PMC9137298

[12] Chu S Wang H. Outcome expectations and older adults with knee osteoarthritis: their exercise outcome expectations in relation to perceived health, self-efficacy, and fear of falling. Healthcare. (2022) 11:57. doi: 10.3390/healthcare11010057, 36611517 PMC9819286

[13] Kubo M Maeda T Kumagai K Amano Y Fujikawa H Isoya E . Phenotypic classification of knee osteoarthritis according to pain mechanisms; A clinical observational study. J Orthop Sci. (2022) 27:672–6. doi: 10.1016/j.jos.2021.03.006, 33965290

[14] Wei X. The relationship between pain catastrophizing in patients with chronic orthopedic pain and pain intensity, as well as emotional state. Chin Clin Nurs. (2021) 13:1–05.

[15] Jia L Fang L Jie Y Xiaowei L Ruonan W. The mediating role of pain catastrophizing in social support and anxiety/depression among patients during hospitalization after total knee arthroplasty. J Bengbu Med Coll. (2022) 47:1746–51. doi: 10.13898/j.cnki.issn.1000-2200.2022.12.029

[16] Yang S Woon EYS Griva K Tan BY. A qualitative study of psychosocial factors in patients with knee osteoarthritis: insights learned from an asian population. Clin Orthop Relat Res. (2023) 481:874–84. doi: 10.1097/CORR.0000000000002526, 36580492 PMC10097569

[17] Aily JB de Almeida AC Ramírez PC Da Silva Alexandre T Mattiello SM. Lower education is an associated factor with the combination of pain catastrophizing and kinesiophobia in patients with knee osteoarthritis? Clin Rheumatol. (2021) 40:2361–7. doi: 10.1007/s10067-020-05518-1, 33230685

[18] Nishigami T Tanaka S Mibu A Imai R Wand BM. Knee-related disability was largely influenced by cognitive factors and disturbed body perception in knee osteoarthritis. Sci Rep. (2021) 11:5835. doi: 10.1038/s41598-021-85307-1, 33712725 PMC7970993

[19] Carriere JS Lazaridou A Martel MO Cornelius M Campbell C Smith M . The moderating role of pain catastrophizing on the relationship between partner support and pain intensity: a daily diary study in patients with knee osteoarthritis. J Behav Med. (2020) 43:807–16. doi: 10.1007/s10865-019-00121-5, 31828692

[20] Matthias MS Hirsh AT Ofner S Daggy J. Exploring the relationships among social support, patient activation, and pain-related outcomes. Pain Med. (2022) 23:676–85. doi: 10.1093/pm/pnab306, 34718764

[21] Faul F Erdfelder E Lang A Buchner A. G*power 3: a flexible statistical power analysis program for the social, behavioral, and biomedical sciences. Behav Res Methods. (2007) 39:175–91. doi: 10.3758/bf03193146, 17695343

[22] Ping N Jingli C Na L. Sample size estimation in quantitative research in nursing studies. Chin J Nurs. (2010) 45:378–80.

[23] Sullivan MJL Bishop SR Pivik J. The pain catastrophizing scale: development and validation. Psychol Assess. (1995) 7:524.

[24] Yining W. Analysis of the Relationship between Pain Catastrophizing and Fatigue in Patients with Inflammatory Joint Diseases and the Mediating Effect of Physical Activity. Anhui Province, China: Anhui Medical University. (2024). doi: 10.26921/d.cnki.ganyu.2024.000341

[25] Dawson J Fitzpatrick R Murray D Carr A. Questionnaire on the perceptions of patients about total knee replacement. J Bone Joint Surg Br. (1998) 80:63–9. doi: 10.1302/0301-620x.80b1.7859, 9460955

[26] Zigmond AS Snaith RP. The hospital anxiety and depression scale. Acta Psychiatr Scand. (1983) 67:361–70. doi: 10.1111/j.1600-0447.1983.tb09716.x, 6880820

[27] Weifei Y Junmian X. The application and evaluation of the "comprehensive hospital anxiety and depression scale" in patients of comprehensive hospitals. Chin J Behav Med. (1993) 2:17–9.

[28] Yuanyuan X. Theoretical basis and research application of the “social support rating scale”. J Clin Psychiatry. (1994) 2:98–100.

[29] Smilkstein G Ashworth C Montano D. Validity and reliability of the family APGAR as a test of family function. J Fam Pract. (1982) 15:303–11. 7097168

[30] Lazaridou A Martel MO Cornelius M Franceschelli O Campbell C Smith M . The association between daily physical activity and pain among patients with knee osteoarthritis: the moderating role of pain catastrophizing. Pain Med. (2019) 20:916–24. doi: 10.1093/pm/pny129, 30016486 PMC6497093

[31] Patel RM Anderson BL Bartholomew JB. Interventions to manage pain catastrophizing following total knee replacement: a systematic review. J Pain Res. (2022) 15:1679–89. doi: 10.2147/JPR.S353385, 35726310 PMC9206032

[32] Chua J Castrejon I Block JA Malfait A-M Pincus T. Fri0549 osteoarthritis (OA) and rheumatoid arthritis (RA) patients have similar disease burdens at first visit to an academic rheumatology setting, but OA patients have a higher burden at a 6-month follow-up visit. Ann Rheum Dis. (2018) 77:800. doi: 10.1136/annrheumdis-2018-eular.5856

[33] Yufei L Yong L Kanghua L. A comparative study of knee osteoarthritis between urban and rural areas in Hunan province. J Hunan Normal Univ. (2015) 12:50–3.

[34] Abramoff B Caldera FE. Osteoarthritis: pathology, diagnosis, and treatment options. Med Clin North Am. (2020) 104:293–311. doi: 10.1016/j.mcna.2019.10.007, 32035570

[35] He C Li Q Huang R Gao X Li L Fan P. Pain phenotype in knee osteoarthritis: implications for mechanism-based therapy. Orthop Surg. (2025) 17:3007–21. doi: 10.1111/os.70161, 40855815 PMC12580230

[36] Lethem J Slade PD Troup JD Bentley G. Outline of a fear-avoidance model of exaggerated pain perception--I. Behav Res Ther. (1983) 21:401–8. doi: 10.1016/0005-7967(83)90009-8, 6626110

[37] Licciardone JC Ibrahim M Baker J Thornton T Vu S. Pain catastrophizing and risk of progression to widespread pain among patients with chronic low back pain: a retrospective cohort study. Musculoskelet Sci Pract. (2024) 69:102886. doi: 10.1016/j.msksp.2023.102886, 38096594

[38] Wideman TH Finan PH Edwards RR Quartana PJ Buenaver LF Haythornthwaite JA . Increased sensitivity to physical activity among individuals with knee osteoarthritis: relation to pain outcomes, psychological factors, and responses to quantitative sensory testing. Pain. (2014) 155:703–11. doi: 10.1016/j.pain.2013.12.028, 24378879

[39] Zhenzhen X. The mediating effect of pain catastrophizing between pain vigilance and pain interference in patients with chronic pain. Front Soc Scie. (2024) 13:142–9. doi: 10.12677/ASS.2024.133197

[40] Wang L Zhang L Yang L Cheng-Qi H. Effectiveness of pain coping skills training on pain, physical function, and psychological outcomes in patients with osteoarthritis: a systemic review and meta-analysis. Clin Rehabil. (2021) 35:342–55. doi: 10.1177/0269215520968251, 33103915

[41] Supe HM Mungikar SS Katage GA Garg KA Wani SK. Effect of pain neuroscience education with conventional physiotherapy via telerehabilitation on pain catastrophizing and function in patients with osteoarthritis knee: a randomized controlled trial. J Midlife Health. (2023) 14:123–9. doi: 10.4103/jmh.jmh_33_23, 38029040 PMC10664057

[42] Rogers AH Farris SG. A meta-analysis of the associations of elements of the fear-avoidance model of chronic pain with negative affect, depression, anxiety, pain-related disability and pain intensity. Eur J Pain. (2022) 26:1611–35. doi: 10.1002/ejp.1994, 35727200 PMC9541898

[43] McHugh RK Kneeland ET Edwards RR Jamison R Weiss RD. Pain catastrophizing and distress intolerance: prediction of pain and emotional stress reactivity. J Behav Med. (2020) 43:623–9. doi: 10.1007/s10865-019-00086-5, 31376099 PMC6995408

[44] Fu K Metcalf B Bennell KL Zhang Y Deveza LA Robbins SR . The association between psychological factors and pain exacerbations in hip osteoarthritis. Rheumatology. (2021) 60:1291–9. doi: 10.1093/rheumatology/keaa494, 32940708

[45] Skelton M Carr E Buckman JEJ Davies MR Goldsmith KA Hirsch CR . Trajectories of depression and anxiety symptom severity during psychological therapy for common mental health problems. Psychol Med. (2023) 53:6183–93. doi: 10.1017/S0033291722003403, 36510471 PMC10520600

[46] Boumparis N Karyotaki E Kleiboer A Hofmann SG Cuijpers P. The effect of psychotherapeutic interventions on positive and negative affect in depression: a systematic review and meta-analysis. J Affect Disord. (2016) 202:153–62. doi: 10.1016/j.jad.2016.05.019, 27262637

[47] Dugas MJ Giguère Marchal K Cormier S Bouchard S Gouin J Shafran R. Pain catastrophizing and worry about health in generalized anxiety disorder. Clin Psychol Psychother. (2023) 30:852–61. doi: 10.1002/cpp.2843, 36807639

[48] Wei L. Research on the relationship between pain catastrophizing and pain outcomes. Hengyang Normal Univ J. (2013) 34:167–70. doi: 10.13914/j.cnki.cn43-1453/z.2013.04.022

