# Correction: Factors associated with pain catastrophizing in patients with knee osteoarthritis: a cross-sectional study

**DOI:** 10.3389/fpubh.2026.1996354

**Published:** 2026-09-29

**Authors:** Lian Qiu, Yulu Yan, Guorui Li, Lin Cui, Xinyu Wang, Yingchun Wu, Xiang Yang

**Affiliations:** 1 School of Nursing, Chengdu Medical College, Chengdu, China; 2 Sichuan Provincial People's Hospital, Chengdu, China; 3 The 944th Hospital of PLA Joint Logistics Support Force, Chengdu, China; 4 General Hospital of Western Theater Command, Chengdu, China

**Keywords:** anxiety, associated factors, knee osteoarthritis, pain catastrophizing, social support

An incorrect grant number “HZAQ-2023-07” was provided for Key Research Base of Philosophy and Social Science in Zigong City in the Funding statement. The correct grant number is “HZAQ-2023-06”.

An incorrect D-W value was included in the “**Outcome**” section, subsection *3.3 “Factors related to pain catastrophizing”*. The correct paragraph reads:

“Hierarchical regression analysis was employed to explore the factors related to pain catastrophizing in patients with knee osteoarthritis. The D-W statistic is 1.870, indicating that the data are independent. The residual scatter plot shows points distributed symmetrically around zero, suggesting that the homoscedasticity assumption is met. The residual P–P plot and residual histogram conform to a normal distribution. The VIF value is lower than 5 and the tolerance value is above 0.2, indicating that there is no serious multicollinearity problem among the variables in the regression model. In Model 1, we used the pain catastrophizing items as the dependent variable and selected variables such as age, average monthly family income, whether using opioid drugs, whether having a caregiver, duration of pain, and NRS Scores (activity) as the independent variables for hierarchical regression analysis, which were statistically significant. After controlling for the general characteristics, we included variables such as Oxford Knee Score, Anxiety, Social Support, Family Care and Depression as independent variables in Model 2. The results of the regression model indicated that NRS Scores (activity), Anxiety, Oxford Knee Score, and Social Support were the relevant factors for pain catastrophizing (**Table 4**).”

The original version of this article has been updated.

